# A Sliced Parabolic Equation Method to Characterize Maritime Radio Propagation

**DOI:** 10.3390/s23104721

**Published:** 2023-05-12

**Authors:** Yuzhen Wang, Ting Zhou, Tianheng Xu, Honglin Hu

**Affiliations:** 1 Shanghai Advanced Research Institute, Chinese Academy of Sciences, Shanghai 201210, China; wangyuzhen2018@sari.ac.cn (Y.W.); xuth@sari.ac.cn (T.X.); huhl@sari.ac.cn (H.H.); 2 School of Electronic, Electrical and Communication Engineering, University of Chinese Academy of Sciences, Beijing 100049, China; 3 School of Microelectronics, Shanghai University, Shanghai 200444, China; 4 Shanghai Frontier Innovation Research Institute, Shanghai 201100, China

**Keywords:** maritime communications, radio propagation measurement, evaporation duct, elevated duct, path loss

## Abstract

For maritime broadband communications, atmospheric ducts can enable beyond line-of-sight communications or cause severe interference. Due to the strong spatial–temporal variability of atmospheric conditions in near-shore areas, atmospheric ducts have inherent spatial heterogeneity and suddenness. This paper aims to evaluate the effect of horizontally inhomogeneous ducts on maritime radio propagation through theoretical analysis and measurement validation. To make better use of meteorological reanalysis data, we design a range-dependent atmospheric duct model. Then, a sliced parabolic equation algorithm is proposed to improve the prediction accuracy of path loss. We derive the corresponding numerical solution and analyze the feasibility of the proposed algorithm under the range-dependent duct conditions. A 3.5 GHz long-distance radio propagation measurement is utilized to verify the algorithm. The spatial distribution characteristics of atmospheric ducts in the measurements are analyzed. Based on actual duct conditions, the simulation results are consistent with the measured path loss. The proposed algorithm outperforms the existing method during the multiple duct periods. We further investigate the influence of different duct horizontal characteristics on the received signal strength.

## 1. Introduction

With the rapid development of marine activities, there has been a growing demand for high-speed and cost-efficient maritime communications [1,2,3,4,5]. To achieve space–air–ground–sea integrated communication networks for sixth-generation (6G) communications, it is essential to build a seamless maritime communication network to extend broadband coverage to the sea area [6,7]. The LTE-Maritime project proved the feasibility of high-speed wireless communication in coastal areas (up to 100 km from a shore) [8]. Unlike terrestrial scenarios, the maritime wireless propagation environment has unique meteorological characteristics, such as sea wave fluctuations [9] and the ducting effect over the sea surface [10]. Current fifth-generation (5G) cellular networks designed for terrestrial scenarios will be heavily affected by these maritime environmental factors [11]. Therefore, it is crucial to establish reliable maritime channel models for maritime wireless networks, especially for near-coast wireless networks [12].

Maritime communications face many application scenarios, such as land-to-ship, ship-to-ship, and buoy-to-ship [13]. Researchers have recently launched several measurement campaigns to investigate wireless maritime channel properties [14]. In particular, recent measurements have shown that evaporation ducts and elevated ducts can greatly reduce the transmission loss [15]. The atmospheric duct is caused by a rapid decrease in atmospheric refractivity over the sea surface [16]. The trapped signal in the duct layer has an extra gain and can travel hundreds of kilometers [17]. In addition, atmospheric ducts have the characteristics of wide areas and long durations. The ducting effect usually causes unexpected remote interference and seriously degrades the performance of coastal 5G networks and LTE networks [18]. More importantly, atmospheric ducts can be utilized to improve wireless coverage and transmission efficiency, especially for land-to-ship communications [19]. It can also improve unmanned aerial vehicle (UAV)-aided maritime communication when the UAV is deployed at the proper height in atmospheric ducts [20].

Therefore, the atmospheric duct effect on maritime radio propagation and channel modeling has drawn growing attention from researchers [21]. Gunashekar et al. measured the 2 GHz maritime channel and analyzed the influence of the antenna height on the ducting effect [22]. The authors in [23] measured a near-shore wireless channel at 5 GHz, where the evaporation duct layer is assumed to be horizontally homogeneous. The authors in [24] conducted a 30 km maritime communication experiment and proposed a ray-tracing model to analyze large-scale fading. In [25], an 87 km maritime microwave measurement demonstrated that atmospheric ducts can enhance the received signal by more than 40 dB and revealed that the evaporation duct communication is feasible. In [26], a 107 km measurement at the C band was conducted in the Bohai Sea and results indicated that the distribution of fast fading is close to a Rayleigh distribution.

These empirical models are completed through a large number of actual measurements and useful for maritime communications. Note that the measurements of maritime radio channels require a great deal of financial and human resources. Such statistical methods are not sufficient for fast variations in dynamic maritime environments [27]. Ray-tracing and parabolic equation methods have better applicability. The authors in [28] proposed a novel ship–ship round earth loss model by incorporating the ray-tracing method and wind speed. A novel three-dimensional non-stationary UAV-to-ship channel model was proposed, which considered the multi-bounce components introduced by atmospheric ducts [29].

Recently, parabolic equation methods have been widely used for radio propagation simulations in maritime environmental conditions [30]. A parabolic equation simulation tool (PETOOL) for evaporation ducts was proposed [31,32], which can support a wide variety of evaporation duct models. Using the propagation loss obtained from PETOOL, a vertically separated MIMO ship-to-ship communication system was proposed [33]. In [34], the authors proposed a large-scale path loss model for the ducting channel. For rough sea surface conditions, an improved parabolic equation method was proposed based on a pade approximation form [35]. The fading characteristics in evaporation ducts were studied with average duct heights in the South China Sea [36]. A ship-to-ship channel model was proposed, which considered the influence of evaporation ducts on the scatterer distribution [37]. A prediction map of radio propagation was proposed with help of atmospheric duct data in the Korean coastal area [38]. The authors in [39] proposed a log-distance maritime wireless channel at 8 GHz, and the meteorological data pointed out that the difference in the evaporation duct profiles at the Tx and Rx increases with increasing link distance. Machine learning has been widely used in the processing of wireless channel measurements [40]. In [41], a two-stage deep learning network was designed to characterize duct effects. In [42], a duct channel model based on a neural network was proposed to predict propagation loss. It should be noted that the training data in [41,42] were generated in ideal evaporation ducts. To improve the maritime communication reliability, the authors in [43] proposed a hybrid method to exploit the combination of cooperative diversity and statistical evaporation duct heights.

In contrast to the homogeneous assumptions in [30,31,32,33,34,35,36,37,38,39,40,41,42,43], the duct layers are range-dependent. The strong spatial–temporal variability of the lower atmosphere in near-shore areas can cause horizontal variability in atmospheric ducts. A measurement campaign in the Indian Ocean and the South China Sea has also shown the horizontal variability of atmospheric ducts [44,45]. It is obvious that disregarding the range-dependent duct environment will lead to inaccurate prediction results [46].

To the best of the authors’ knowledge, most existing models cannot reflect the effect of horizontally inhomogeneous ducts. The range-dependent characteristics should be taken into consideration. The decision methods of maritime communication with duct conditions are also lacking.

With the development of Maritime Internet of Things (M-IoT), future maritime wireless communications need to utilize meteorological information to establish sensitive maritime channels that cope with changing environments [47]. Moreover, the state-of-the-art numerical weather prediction can provide data of high spatial–temporal resolution. This also motivates us to explore a framework of meteorological information-aided maritime radio propagation characterization. Our preliminary study [48] presents a basic environmental information-aided interference simulation method and verifies the feasibility of meteorological data.

In this paper, we aim to study the effect of horizontally inhomogeneous ducts on maritime radio propagation. Considering the limitations of the existing methods, we propose a sliced parabolic equation algorithm to predict radio propagation characteristics in atmospheric ducts. The major contributions are summarized as follows.

- We investigate existing atmospheric duct models and their effects on maritime radio propagation. Subsequently, we propose a range-dependent duct model to support the actual duct environment. The spatial–temporal characteristics of atmospheric ducts are studied by using the meteorological reanalysis data.
- We propose a sliced parabolic equation algorithm to improve the prediction accuracy of path loss. Moreover, we derive the corresponding numerical solution and the approximate error expression in inhomogeneous environments.
- We validate the algorithm’s effectiveness by the 3.5 GHz field measurement with the actual meteorological data. Compared with the traditional method, the proposed algorithm has higher accuracy during the multiple duct periods.
- We evaluate the proposed algorithm in various range-dependent ducts, including evaporation ducts and elevated ducts. Simulation results show that the duct’s horizontal characteristics have a significant impact on path loss.

The rest of the paper is organized as follows. In Section 2, maritime radio propagation scenarios and three basic duct models are introduced. Based on a range-dependent duct model, Section 3 presents a sliced parabolic equation algorithm. The numerical solution and feasibility derivation are also provided. In Section 4, the effectiveness of the proposed algorithm is verified by the 3.5 GHz field measurements. Section 5 presents the simulation results in evaporation ducts and elevated ducts with different horizontal characteristics. Finally, conclusions are given in Section 4.

## 2. System Model and Preliminaries

### 2.1. Maritime Radio Propagation Scenarios

**Figure 1:**
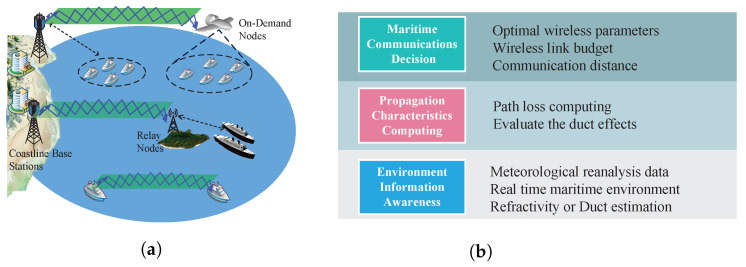
(**a**) Typical propagation scenarios in near-coast maritime communications, which can be affected by atmospheric ducts. (**b**) The framework of meteorological information-aided radio propagation characterization for maritime communications.

A typical near-shore maritime communication network is shown in Figure 1, where various wireless communication links are adopted to provide wide coverage and a cost-efficient broadband data service [49]. In general, a near user group can be directly served by the coastline base station (CBS). Relay nodes or on-demand nodes such as UAVs are utilized for coverage enhancement. The CBS also serves on-demand wireless nodes. Ship-to-ship communications aim to provide high rate transmission, such as offshore device-to-device communication [50].

As the near-shore maritime communication network will play increasingly important roles, the atmospheric duct is a non-negligible factor that will significantly impact the performance of maritime wireless systems, especially between 2 GHz and 20 GHz [15]. Atmospheric ducts usually exist above the sea surface and in coastal regions. As shown in Figure 1, the captured signal in the atmospheric ducts could propagate beyond the line of sight. Atmospheric ducts will affect these wireless links, including CBS-to-ship/relay, ship-to-ship, and CBS-to-UAV channels. The utilization of atmospheric ducts is still in the early stage.

### 2.2. Descriptions of Atmospheric Duct Models

The formation of atmospheric ducts depends on atmospheric refractive conditions. Without loss of generality, the refractivity *N* is defined as [51]

(1) $$N=\left(n-1\right)\times 10^{6}=\frac{77.6}{T}\left(P+\frac{4810e}{T}\right)\;N-\mathrm{units},$$

where *P* is the atmospheric pressure (hPa), *T* denotes the absolute temperature (K), *e* is the water vapor pressure (hPa) and *n* is the atmospheric refractive index.

The curvature of the Earth needs to be considered in wireless channel modeling. The modified refractivity *M* is defined as

(2) $$M\left(z\right)=N\left(z\right)+\left(\frac{z}{R_{e}}\right)\times 10^{6}\;M-\mathrm{units},$$

where *z* is the height above the surface level in km and $R_{e}$ denotes the Earth’s radius, whose average value is approximately 6,371,000 m.

Based on (2), the modified refractivity gradient is written as

(3) $$\frac{dM}{dz}=\frac{dN}{dz}+0.157.$$

**Figure 2:**
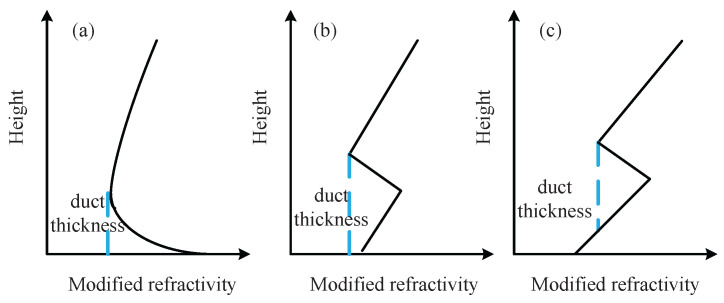
Modified refractivity profiles of atmospheric ducts. (**a**) Evaporation duct. (**b**) Surface-based duct. (**c**) Elevated duct.

The trapping condition is associated with a negative modified refractivity gradient. The duct height is given by the height where the gradient of the modified refractivity changes from negative to positive. The radio signals can be effectively trapped between the surface and ducting layer. As shown in Figure 2, there are three main types of atmospheric ducts: evaporation ducts, surface-based ducts, and elevated ducts.

Evaporation ducts are the most common type in maritime environments. This is caused by the rapid decrease in humidity with height and high water evaporation. The probability of occurrence is approximately 90% in equatorial and tropical regions [30]. The Red Sea and the Persian Gulf are permanent duct areas. The average duct heights are mostly between 10 m and 40 m [52]. Evaporation ducts can affect the stability of CBS-to-ship and ship-to-ship links. Due to their high availability, evaporative ducts are the most promising type for maritime communication applications.

For maritime environments, the modified refractivity of evaporation ducts can be expressed by a widely accepted logarithmic function [53]

(4) $$M\left(z\right)=M_{0}+0.125z-0.125\delta \ln\left(\frac{z+z_{0}}{z_{0}}\right),$$

where $M_{0}$ is the value of modified refractivity at the surface, δ is the evaporation duct height, and $z_{0}$ is the aerodynamic roughness length, which can be assumed to be $1.5\times 10^{-4}\;m$.

Elevated ducts are caused by the presence of marine layers and mainly occur along the trade winds and close to the coastline. Surface-based ducts are special cases of elevated ducts. The occurrence rate of elevated ducts can be up to 50%. The elevated duct height can reach hundreds of meters. Therefore, elevated ducts can affect the CBS-to-UAV/relay links and cause unexpected remote interference in cellular networks. The profile of elevated ducts can be modeled with a trilinear curve, and can be written as [54]

(5) $$M\left(z\right)=M_{0}+\left\{\begin{array}{cc} g_{1}z, & z\leq h_{1},\\ g_{1}h_{1}+g_{2}\left(z-h_{1}\right), & h_{1}<z\leq h_{2},\\ g_{1}h_{1}+g_{2}\left(h_{2}-h_{1}\right)+g_{3}\left(z-h_{2}\right), & z>h_{2} \end{array}\right.$$

where $g_{1}$, $g_{2}$, and $g_{3}$ are the slopes of the modified refractivity.

### 2.3. Radio Propagation Characteristics

Maritime radio propagation simulation requires modeling of atmospheric parameters and complex boundary problems. Based on the parabolic approximation of the Helmholtz wave equation, the parabolic equation methods could model the changes in refractivity in the atmosphere and simulate complex boundary conditions [35]. Moreover, the parabolic equation methods also have the potential to calculate radio propagation in complex and large-scale maritime environments.

With parabolic equation methods and atmospheric parameters, the path loss can be written as [53]

(6) PL=−20log|u(x,z)|+20log(4π)+10log(x)−30log(λ),

where *u* denotes the field strength computed by parabolic equation methods, *x* is the distance, and λ denotes the wavelength.

The strong spatial and temporal variability of the lower atmosphere in coastal and near-shore areas can cause horizontal variability in atmospheric ducts. However, the prediction accuracy may be lost due to the assumption of horizontal homogeneity in the traditional parabolic equation methods. As shown in Figure 1, it is important to study a framework of meteorological information-aided radio propagation characterization, from maritime environment information awareness to the deployment decision. Therefore, an accurate and general algorithm with the help of meteorological reanalysis products is essential to deal with range-dependent atmospheric ducts.

## 3. Sliced Parabolic Equation Algorithm in Horizontally Inhomogeneous Duct Environments

In this section, we present a range-dependent duct model to make better use of meteorological reanalysis data. Then, we propose a sliced parabolic equation algorithm to simulate electromagnetic wave propagation in the maritime environment. The numerical solution and feasibility derivation are also provided. In the remainder of this section, we will present the workflow and implementation details.

### 3.1. Range-Dependent Duct Model

In this subsection, we will introduce the range-dependent refractivity and duct environments.

In the ideal case, assume that the meteorological data at different heights and distances can be measured by microwave refractometers and lidar. The refractivity N(x,z) can be defined as

(7) $$N\left(x,z\right)=\frac{77.6}{T(x,z)}\left(P\left(x,z\right)+\frac{4810e(x,z)}{T(x,z)}\right)\;N-\mathrm{units}.$$

However, these methods are high-cost. On the other hand, the fast developments of mesoscale numerical weather prediction can provide meteorological data with a high spatial and temporal resolution and make it possible to analyze the horizontal heterogeneity of ducts. It is possible to estimate the refractivity index and duct heights from standard meteorological data. Specifically, meteorological measurements can provide the temperature, humidity, wind speed, pressure, and sea surface temperature at certain heights. The environmental information is used as input parameters. Then, the profiles of temperature, humidity, and air pressure can be estimated with the Monin–Obukhov similarity theory [55], and the refractivity profiles can be obtained by (7). Finally, duct heights can be estimated.

For example, the fifth-generation European Center for Medium-Range Weather Forecasts (ECMWF) reanalysis data (ERA5) provide hourly products of meteorological variables. Its horizontal resolution is 0.25°. Therefore, we aim to use the up-to-date meteorological reanalysis product to study the spatial and temporal distributions of the duct environment.

**Figure 3:**
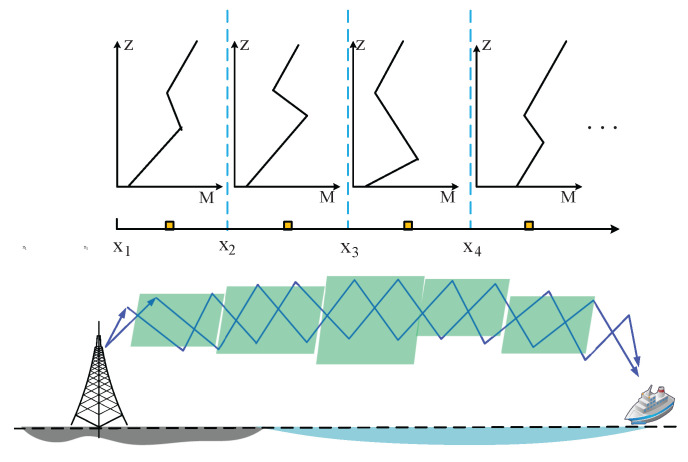
The range-dependent duct model. The yellow squares represent the locations of meteorological measurements.

Therefore, we adopt a range-dependent duct model, considering the actual horizontal resolution of meteorological data and computational accuracy. As shown in Figure 3, we assume that there are *L* slices of different atmospheric vertical structures in the horizontal direction. In each slice, N(x,z) is almost constant in the x direction. The formulas of the range-dependent duct environments are as follows:

(8) $$\begin{array}{cc} \; & M_{slice-1}\left(x,z\right)=N\left(\frac{x_{1}+x_{2}}{2},z\right)+\left(\frac{z}{a}\right)\times 10^{6},\;\left(x_{1}<x\leq x_{2}\right),\\ \; & M_{slice-2}\left(x,z\right)=N\left(\frac{x_{2}+x_{3}}{2},z\right)+\left(\frac{z}{a}\right)\times 10^{6},\;\left(x_{2}<x\leq x_{3}\right),\\ \; & M_{slice-3}\left(x,z\right)=N\left(\frac{x_{3}+x_{4}}{2},z\right)+\left(\frac{z}{a}\right)\times 10^{6},\;\left(x_{3}<x\leq x_{4}\right). \end{array}$$

### 3.2. Sliced Parabolic Equation Algorithm

In the rectangular coordinate system, the wave equation satisfies the approximation of the Helmholtz equation,

(9) $$\frac{\partial^{2}\psi }{\partial x^{2}}+\frac{\partial^{2}\psi }{\partial z^{2}}+k_{0}^{2}n{\left(x,z\right)}^{2}\psi =0,$$

where $k_{0}=2\pi /\lambda$ denotes the wavenumber in a vacuum, *n* is the refractive index of the tropospheric medium, *x* is the propagation distance, and *z* is the height. The refractive index n(x,z) varies with distance and height.

Substituting the simplified function $u\left(x,z\right)=e^{-ik_{0}x}\psi \left(x,z\right)$, the field u(x,z) is expressed as

(10) $$\begin{array}{cc} \; & \frac{\partial^{2}u\left(x,z\right)}{\partial x^{2}}+2ik_{0}\frac{\partial u(x,z)}{\partial x}\\ \; & +\frac{\partial^{2}u\left(x,z\right)}{\partial z^{2}}+k_{0}^{2}\left(n{\left(x,z\right)}^{2}-1\right)u\left(x,z\right)=0. \end{array}$$

The differential operator in (10) can be factored in terms of two pseudo-differential operators [35]. Then, the above equation can be rewritten as

(11) $$\left[\frac{\partial }{\partial x}+ik_{0}\left(1-Q\right)\right]\left[\frac{\partial }{\partial x}+ik_{0}\left(1+Q\right)\right]u=0,$$

where *Q* is expressed as

(12) $$Q=\sqrt{\frac{1}{k_{0}^{2}}\frac{\partial^{2}}{\partial z^{2}}+n^{2}\left(x,z\right)}.$$

The forward propagation electromagnetic waves are expressed as

(13) $$\frac{\partial u}{\partial x}=-ik_{0}\left(1-Q\right)u.$$

The approximate forms of the operator *Q* determine the scope of the elevation angle and the accuracy of calculation. Specifically, by defining $\varepsilon =n^{2}\left(x,z\right)-1$, $\mu =\frac{1}{k_{0}^{2}}\frac{\partial^{2}}{\partial z^{2}}$, *Q* is given by $Q=\sqrt{1+\varepsilon +\mu }$. The pseudo-differential operator of the Feit–Fleck type is given by

(14) $$Q\approx \sqrt{1+\mu }+\sqrt{1+\varepsilon }-1.$$

Then, (13) can be rewritten as

(15) $$\begin{array}{c} \frac{\partial u(x,z)}{\partial x}=ik_{0}\left[\sqrt{1+\frac{1}{k_{0}^{2}}\frac{\partial^{2}}{\partial z^{2}}}\right]u+ik_{0}\left[n\left(x,z\right)-2\right]u\left(x,z\right). \end{array}$$

The wide-angle parabolic equation of the Feit–Fleck type can be expressed as

(16) $$\frac{\partial u(x,z)}{\partial x}=\left[i\sqrt{k_{0}^{2}+\partial^{2}/\partial z^{2}}+ik_{0}\left(n\left(x,z\right)-2\right)\right]u\left(x,z\right).$$

The formal solution can be formulated as

(17) $$\begin{array}{c} u\left(x+\Delta x,z\right)=e^{\int_{x}^{x+\Delta x}\left[i\sqrt{k_{0}^{2}+\partial^{2}/\partial z^{2}}+ik_{0}\left(n\left(x,z\right)-2\right)\right]dx}u\left(x,z\right). \end{array}$$

The split-step Fourier transform technique is widely used for parabolic equation’s numerical solutions. It requires a large amount of computational power when n(x,z) varies with distance.

In the *L*-th slice, we define $A=i\sqrt{k_{0}^{2}+\partial^{2}/\partial z^{2}},B=ik_{0}\left(n\left(x,z\right)-2\right)$, and the temporary variable is expressed as

(18) Ω=B+A.

Next, we aim to solve the differentiation equation, which can be formulated as

(19) $$\begin{array}{c} u\left(x+\Delta x,z\right)=e^{\int_{x}^{x+\Delta x}\Omega \left(x,z\right)dx}u\left(x,z\right). \end{array}$$

By applying the integral mean-value theorem in the i-th slice, it has an approximate formula as

(20) $$\int_{x}^{x+\Delta x}\Omega dx=\left(B+A\right)\Delta x.$$

Therefore, the general solution of the differentiation equation is given by

(21) $$u\left(x+\Delta x,z\right)=e^{(B+A)\Delta x}u\left(x,z\right).$$

Then, the pseudo-differential operator is formulated as

(22) $$e^{(B+A)\Delta x}=e^{A\Delta x}\;\cdot \;e^{B\Delta x}.$$

Define $v\left(x,z\right)=e^{A\Delta x}u\left(x,z\right)$. The field u(x,z) can be expressed as follows:

(23) $$u\left(x+\Delta x,z\right)=v\left(x,z\right)e^{ik_{0}\left(n\left(x,z\right)-2\right)\Delta x}.$$

By using Taylor expansion, $v\left(x,z\right)=e^{A\Delta x}u\left(x,z\right)$ can be expressed as

(24) $$\begin{array}{cc} \; & v\left(x,z\right)\\ \; & =\left[1+i\Delta x\sqrt{k_{0}^{2}+\frac{\partial^{2}}{\partial z^{2}}}-\frac{1}{2}{\left(\Delta x\right)}^{2}\left(k_{0}^{2}+\frac{\partial^{2}}{\partial z^{2}}\right)+\ldots \right]u\left(x,z\right). \end{array}$$

The Fourier transform of *u* is defined as

(25) $$\begin{array}{c} U\left(x,p\right)=F\left[u\left(x,z\right)\right]=\int_{-\infty }^{+\infty }u\left(x,z\right)e^{-ipz}dz. \end{array}$$

The inverse Fourier transform is given by

(26) $$\begin{array}{c} u\left(x,z\right)=F^{-1}\left[U\left(x,p\right)\right]=\frac{1}{2\pi }\int_{-\infty }^{+\infty }U\left(x,p\right)e^{izp}dp. \end{array}$$

Then, we have $F\left(\sqrt{k_{0}^{2}+\frac{\partial^{2}}{\partial z^{2}}}u\right)=\sqrt{k_{0}^{2}-p^{2}}U\left(x,p\right)$.

We apply the Fourier transform to both sides of (24) and have

(27) $$\begin{array}{cc} \; & V\left(x,p\right)\\ \; & =\left[1+i\Delta x\sqrt{k_{0}^{2}-p^{2}}-\frac{1}{2}{\left(\Delta x\right)}^{2}\left(k_{0}^{2}-p^{2}\right)+\ldots \right]U\left(x,p\right)\\ \; & ≅e^{i\Delta \sqrt{k_{0}^{2}-p^{2}}}U\left(x,p\right). \end{array}$$

The inverse Fourier transform of *v* is obtained as

(28) $$v\left(x,z\right)=F^{-1}\left[e^{i\Delta x\sqrt{k_{0}^{2}-p^{2}}}U\left(x,p\right)\right].$$

By multiplying the refractive index term, the algorithm solution in the current slice is expressed as

(29) $$u\left(x+\Delta x,z\right)=e^{ik_{0}\left(n\left(x,z\right)-2\right)\Delta x}F^{-1}\left[e^{i\Delta x\sqrt{k_{0}^{2}-p^{2}}}U\left(x,p\right)\right].$$

The initial field at the starting position (x=0) is determined by the parameters of the antenna pattern. The initial field can be obtained by fast Fourier transform based on the aperture field and beam pattern. We use the Fourier shift theorems to embody the antenna height and elevation angle. Therefore, the key parameters of the antenna pattern are the height $z_{a}$, the 3dB beamwidth $\theta_{BW}$, and the elevation angle $\theta_{\mathrm{tilt}.\;}$, respectively. The initial field in the *p* domain can be defined as [31]

(30) $$U\left(0,p\right)=f\left(p\right)\exp\left(-ipz_{a}\right)-f^{*}\left(-p\right)\exp\left(ipz_{a}\right).$$

The initial field in the spatial z-domain is computed by taking the inverse fast Fourier transform. The Gaussian antenna pattern can represent various antenna types.

In this paper, the horizontally polarized Gaussian antenna pattern can be defined as

(31) $$f\left(p\right)=\exp\left[-p^{2}w^{2}/4\right],$$

where $w=\frac{\sqrt{2\ln2}}{k\sin\left(\theta_{BW}/2\right)}$. The elevation angle is introduced by shifting the antenna pattern, i.e., $f\left(p\right)\to f\left(p-k\sin\theta_{\mathrm{tilt}\;}\right)$.

At each step, $e^{ik_{0}\left(n\left(x,z\right)-2\right)\Delta x}$ reflects the refraction effect, and $e^{i\Delta x\sqrt{k^{2}-p^{2}}}$ reflects the diffraction effect. Note that $\partial (n{\left(x,z\right)}^{2})/\partial x\approx 0$ is assumed in the derivation of the numerical solution. It is essential to analyze the errors caused by the change in refractive index over distance in dynamic maritime environments.

### 3.3. Feasibility Analysis

In this subsection, we derive the approximate error expressions by the following theorems. With the Taylor expansion, we derive the actual field *u*, which can be expressed as

(32) $$\begin{array}{cc} \; & u{\left(x+\Delta x,z\right)}_{actual}\\ \; & =u+\Delta x\frac{\partial u}{\partial x}+\frac{{\left(\Delta x\right)}^{2}}{2}\frac{\partial^{2}u}{\partial x^{2}}+\frac{{\left(\Delta x\right)}^{3}}{6}\frac{\partial^{3}u}{\partial x^{3}}+\ldots \\ \; & \approx \left[1+\Omega \Delta x+\frac{{\left(\Delta x\right)}^{2}}{2}\left(\frac{\partial \Omega }{\partial x}+\Omega^{2}\right)\right.\\ \; & \left.+\frac{{\left(\Delta x\right)}^{3}}{6}\left(\frac{\partial^{2}\Omega }{\partial x^{2}}+2\frac{\partial \Omega }{\partial x}\Omega +\Omega \frac{\partial \Omega }{\partial x}+\Omega^{3}\right)\right]u\left(x,z\right), \end{array}$$

where $\frac{\partial u}{\partial x}=\Omega u$, and $\frac{\partial \Omega }{\partial x}=\frac{\partial A}{\partial x}+\frac{\partial B}{\partial x}=ik_{0}\frac{\partial n(x,z)}{\partial x}$.

According to the Monin–Obukhov similarity theory and meteorological accuracy, meteorological conditions in the horizontal direction are consistent in a certain distance. In each slice, we assume that the refractivity profile does not change with range, $\frac{\partial n(x,z)}{\partial x}\approx 0$. The field at x+Δx can be expressed as

(33) $$\begin{array}{c} u{\left(x+\Delta x,z\right)}_{ideal}\approx \left[1+\Omega \Delta x+{\left(\Delta x\right)}^{2}\Omega^{2}/2+{\left(\Delta x\right)}^{3}\Omega^{3}/6\right]u\left(x,z\right). \end{array}$$

By comparing $u_{actual}$ with $u_{ideal}$, the relative error caused by the operator *M* can be written as

(34) $$\begin{array}{c} e_{n}=\frac{{\left(\Delta x\right)}^{2}}{2}\frac{\partial \Omega }{\partial x}+\frac{{\left(\Delta x\right)}^{3}}{6}\;\cdot \;\left(\frac{\partial^{2}\Omega }{\partial x^{2}}+2\frac{\partial \Omega }{\partial x}\Omega +\Omega \frac{\partial \Omega }{\partial x}\right)+\ldots \end{array}$$

It can be seen that the prediction error of the wide-angle operator mainly depends on the step size Δx and $\frac{\partial n(x,z)}{\partial x}$. For horizontally inhomogeneous environments, n(x,z) is almost constant in the x direction in each slice, i.e., $\frac{\partial \Omega }{\partial x}\approx 0$ and $\frac{\partial^{2}\Omega }{\partial x^{2}}\approx 0$. Thus, $e_{n}\approx 0$. Therefore, the propagation simulation of the whole path can be completed through the calculation of each slice.

### 3.4. Workflow of the Proposed Algorithm

**Figure 4:**
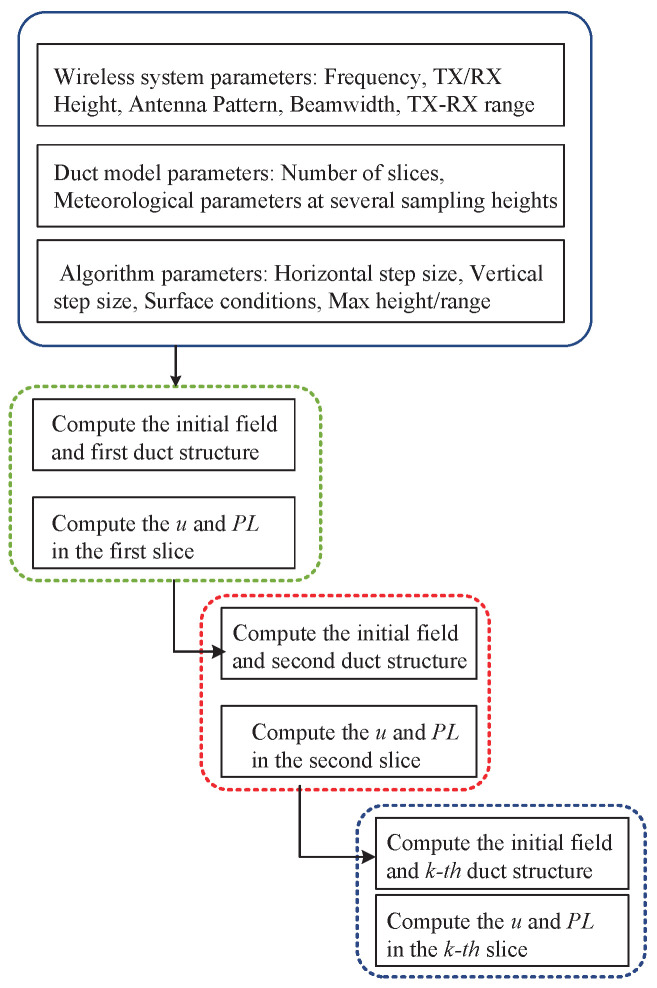
The workflow of the sliced parabolic equation algorithm.

The workflow of the sliced parabolic equation algorithm is shown in Figure 4.

In the first step, the input parameters consist of three parts. (1) We need to set the wireless system parameters, including the frequency, antenna height, antenna pattern, beamwidth, and link distance. (2) Algorithm parameters include the max height, max range, horizontal step size, vertical step size, and surface conditions. (3) For the range-dependent duct model, the number of slices depends on the horizontal resolution of the meteorological data. The refractivity profiles of each slice can be directly provided from the reanalysis data, such as the duct height and duct strength. The refractivity profile can also be obtained by a bulk method based on the Monin–Obukhov similarity theory and flux–profile relationship [48]. The refractivity profile can be computed from the sea surface temperature, wind speed, air temperature, relative humidity, and atmospheric pressure at several sampling heights.

In the second step, we use the proposed algorithm to compute the field strength and path loss in each slice. In the first slice, the initial field strength is based on the antenna pattern. The initial field strength of the second slice is based on the end of the first slice. The full procedure is summarized in Algorithm 1.
**Algorithm 1.** Sliced Parabolic Equation Algorithm.Input:Numerical solution parameters of the proposed algorithm,Wireless system parameters,Duct model parameters.Output: Path loss distribution.Initialize atmospheric vertical structures of *L* slices.for each slice do       Initialize U(0,jΔp) and u(0,mΔz) based on (30)–(31).    for each step size do          Compute V(x+Δx,jΔp) by (27).          Compute v(x+Δx,mΔz) by (26).          Compute $e^{ik_{0}\left(n\left(x,z\right)-2\right)\Delta x}$ in the *L*-th slice.          Update u(x+Δx,mΔz) by (29).    endendObtain the path loss distribution in the whole computational domain based on (6).

## 4. Field Measurement and Verification

In this section, the accuracy of the proposed algorithm will be verified by 3.5 GHz field measurement results. We first give a brief description, and then we discuss the distribution characteristics of the atmospheric ducts by analyzing the meteorological data. The comparison between the measured and simulated path loss is given in the remainder of this section.

### 4.1. Measurement Scenario

Two microwave communication links were set up in the near-shore region, and the radio signal strength was recorded from September 2013 to November 2016. The radio measurement campaign was conducted by the Radio Communications Agency Netherlands. The 3.5 GHz band is very important for the 5G wireless communication system. Therefore, the measurement frequency is set to the 3.5 GHz band.

**Figure 5:**
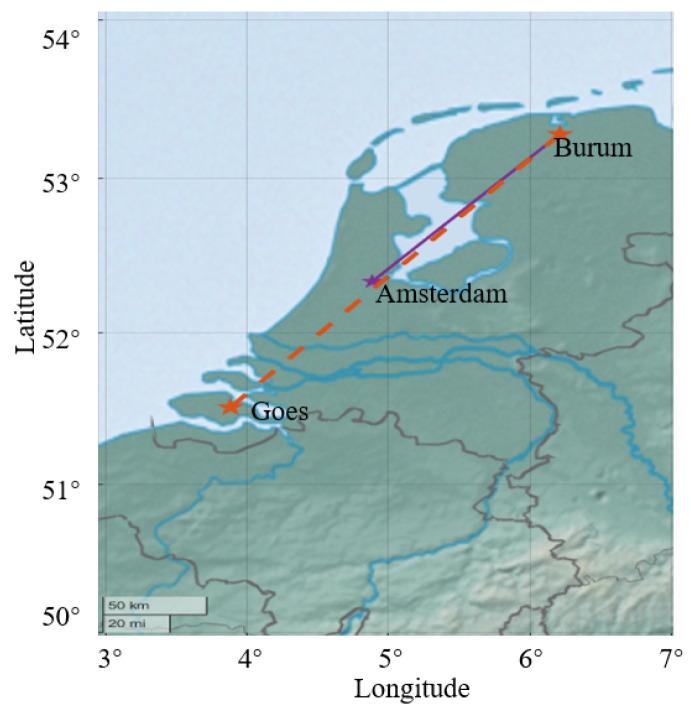
The geographic information of the two wireless links.

Figure 5 illustrates the geographic information of the two wireless links. The receiver is located at 53.282° N, 6.214° E, in the Netherlands. The transmitter at Goes is located at 51.511° N, 3.884° E. The transmitter at Amsterdam is located at 52.336° N, 4.887° E. The two fixed wireless links are the 138 km Amsterdam–Burum path and the 253 km Goes–Burum path. These links are located in coastal areas and river deltas. The terrain is very flat in the experimental zone. The parameters of the antenna height and frequency are listed in Table 1.

The path loss of the 253 km link is approximately 220 dB under normal propagation conditions. On the other hand, the measurement system was able to cope with the path loss (approximately 150 dB) due to atmospheric ducts. Therefore, the upper and lower limits of the signal strength have been considered in the measurement system realization. A more detailed setup can be found in [16]. The RX/TX antenna gain is approximately 26.1 dBi. The vertical 3 dB beamwidth is 8°. This measurement work improved the 3.5 GHz propagation prediction models and can be applied for 5G deployment. Moreover, the measurement results indicated that short-term abnormal propagation caused by atmospheric ducts exists.

In this paper, we will focus on the effect of range-dependent atmospheric ducts on the radio wave propagation. Note that post-measurement corrections and calibrations were performed. To obtain accurate path loss data, the antenna gains and system losses were removed.

### 4.2. Distribution Characteristics of Atmospheric Ducts

First, we aimed to characterize the spatial variability of the atmospheric ducts in the experimental zone. We downloaded synchronous meteorological data from the ERA5 atmospheric reanalysis. It can provide the sea surface temperature, 2 m temperature, 10 m wind speed, 2 m relative humidity, and pressure. It provides the duct top height, the duct base height, and the minimum vertical gradient of refractivity inside the duct layer. The horizontal resolution of ERA5 is 0.25°×0.25° (approximately 30 km).

**Figure 6:**
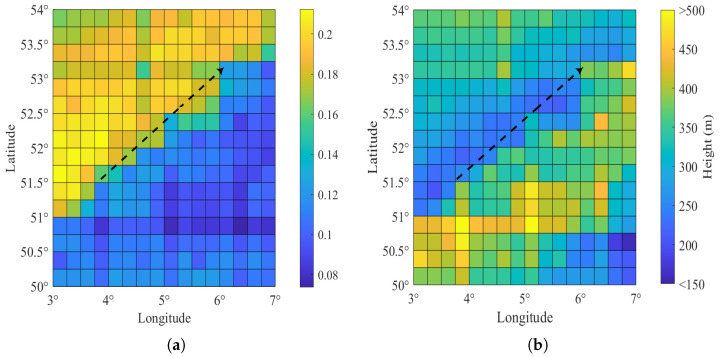
Distribution characteristics of atmospheric ducts. (**a**) Atmospheric duct probability in the experimental zone for the period of 2013–2016. The color represents the probability of occurrence. (**b**) Mean top height of the ducting layer in the experimental zone for the period of 2013–2016. The color represents the mean top height of the ducting layer.

Figure 6 shows the distribution characteristics of the atmospheric ducts in the experimental zone for the period of 2013–2016. Figure 6a illustrates the atmospheric duct probability of the experimental zone. The color represents the probability of duct occurrence. For the wetland-to-sea path (the black line), the coastal zone has a higher probability of duct occurrence. This is because the eastern part of the ocean and the western part of the continent in the trade wind belt are high-probability zones of atmospheric ducts.

The above analysis can be used to identify signal enhancements caused by atmospheric ducts that last for more than three hours. In fact, atmospheric ducts occur more than 50% of the time in many areas, including the Red Sea, the Persian Gulf, and the South China Sea.

Figure 6b shows the mean top heights of atmospheric ducts from September 2013 to November 2016. The color represents the mean top height. The horizontal inhomogeneity of atmospheric ducts is significant in coastal regions. The top heights of the atmospheric ducts in the coastline area are distributed between 200 and 400 m. In coastal areas, the mean top heights of the ducting layer vary significantly. This indicates that the strong spatial and temporal variability of the lower atmosphere in coastal and near-shore areas can cause horizontal variability in the ducting layer. Therefore, the effect of range-dependent atmospheric ducts should be considered carefully if the wireless link is along different latitudes.

### 4.3. Comparison with Measurements

To further verify the effectiveness of the sliced parabolic equation algorithm, the comparison between the measured and simulated path loss is given in this subsection.

In order to be consistent with the time resolutions of meteorological data, we select the hourly measurement data, such as the path loss at 02:00. The spatial resolutions of slices are based on the meteorological resolution. The refractivity conditions are based on the reanalysis meteorological data grid points along the propagation link. Therefore, the Goes–Burum path is divided into seven slices and the Amsterdam–Burum path is divided into four slices based on the meteorological resolution of the ERA5 data.

**Figure 7:**
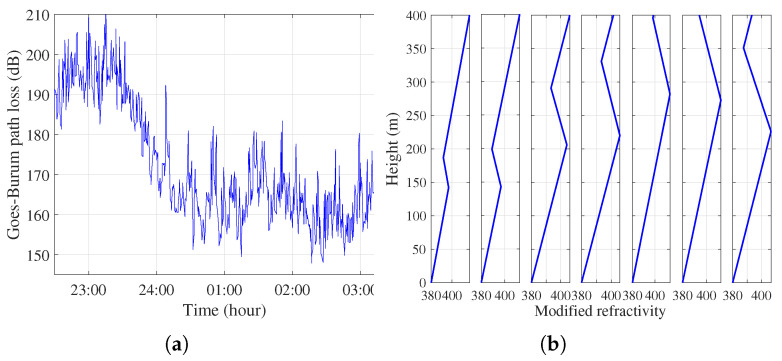
(**a**) Goes–Burum path loss data from 26 August to 27 August 2016. Atmospheric ducts can occur for up to three hours. (**b**) The refractivity conditions of the Goes–Burum path at 02:00.

Figure 7a shows the Goes–Burum path loss data from August 26th to August 27th, 2016. The path loss of the 253 km link is approximately 220 dB under normal propagation conditions. Atmospheric ducts can occur for up to three hours and the signals were received with approximately 50 dB enhancement. Note that the signal enhancement lasted for approximately 3 hours. An example of the refractivity M-profiles of the Goes–Burum path is plotted in Figure 7b. The Goes–Burum path is divided into seven slices. Each distance is approximately 31 km. This indicates the appearance of a range-dependent elevated duct. It is obvious that the atmospheric ducts increase with range.

In this paper, we evaluate the proposed algorithm with the following two baselines: (a) the measured path loss and (b) the widely accepted parabolic equation method presented in references [32,34,35,36,48], which is based on an ideal homogeneous assumption (only using the refractivity profile near the TX/RX).

The dates of significant signal enhancements caused by atmospheric ducts are listed in Table 2. The input parameters of the simulation are listed in Table 3. These simulation parameters and hourly range-dependent refractivity profiles are provided to the algorithm to simulate path loss over the entire height and range. We mainly selected the time period of the occurrence of atmospheric ducts. The discriminant method indicates that the path loss is reduced by more than 40 dB compared to the average path loss. Atmospheric attenuation at 3.5 GHz is small and can be neglected.

**Figure 8:**
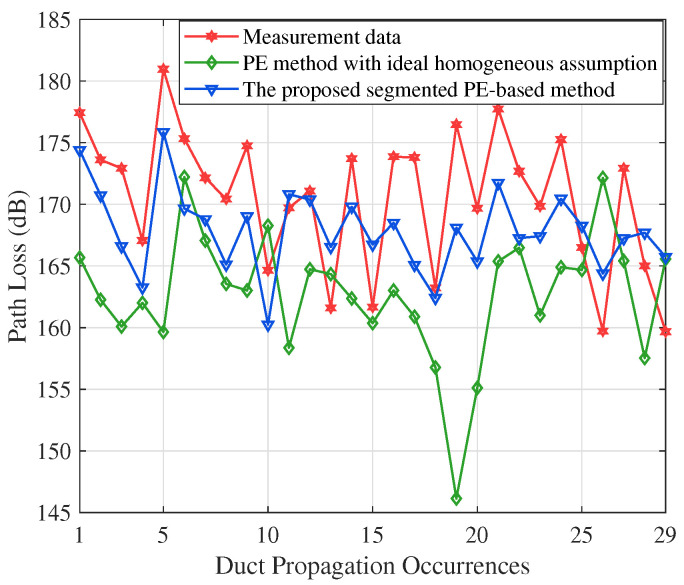
Performance comparison on the Goes–Burum path. The proposed algorithm reduces the average prediction error by approximately 5.01 dB.

Figure 8 shows an hourly path loss comparison of the 253 km Goes–Burum link. The transmitting antenna height is 75 m and the receiver height is 6 m. When an atmospheric duct occurs, the path loss is approximately 170 dB at 253 km. Compared with the multiple measured path loss, the average prediction error of the traditional parabolic equation method is 9.4427 dB. However, the average prediction error of our proposed algorithm is 4.4286 dB. The proposed algorithm shows a good match with the measured path loss. The reasons are as follows. The duct condition on the propagation path varies widely. The sliced parabolic equation algorithm can reflect the inhomogeneity of the propagation path. However, the homogeneous assumption exaggerates the duct effect. Therefore, the effect of range-dependent ducts on electromagnetic wave propagation is significant and should be considered in coastal areas.

**Figure 9:**
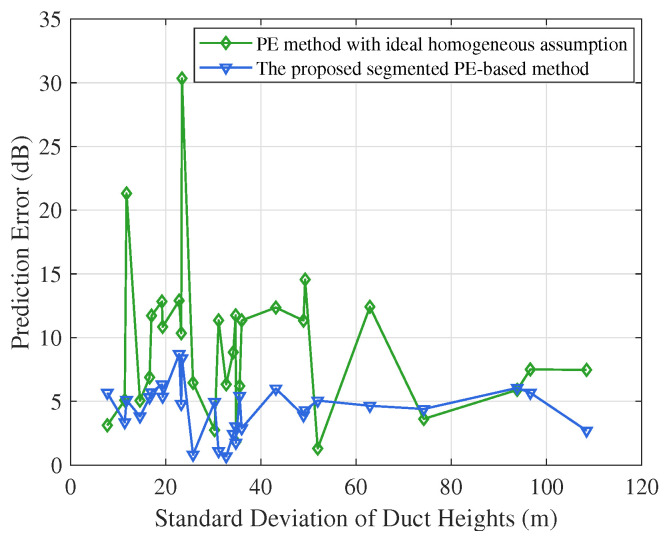
Path loss prediction error vs. standard deviation of duct heights on the Goes–Burum path.

Based on the above simulation results, we compute the standard deviation of the duct heights of seven segments along the Goes–Burum path. Figure 9 shows the prediction error under different standard deviations. We can observe that the proposed algorithm achieves robust performance, while the performance of the traditional parabolic equation method varies widely. This is because the traditional method considering the homogeneous assumption is inaccurate and unstable in actual and dynamic atmospheric ducts.

**Figure 10:**
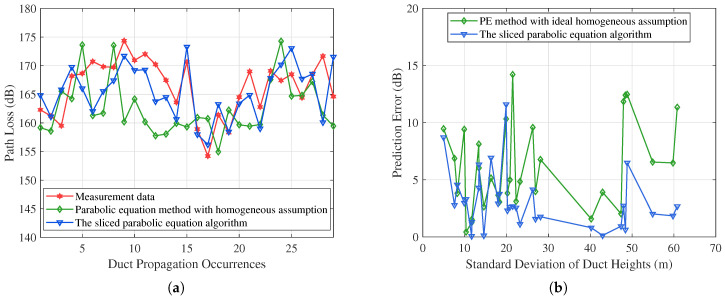
(**a**) Performance comparison on the Amsterdam–Burum path. The proposed algorithm reduces the average prediction error by approximately 3.06 dB. (**b**) Path loss prediction error vs. standard deviation of duct heights on the Amsterdam–Burum path.

Figure 10a compares the prediction performance on the 138 km Amsterdam–Burum path. The path loss under standard atmospheric conditions is approximately 202.31 dB. When the atmospheric duct occurs, the path loss is approximately 165 dB. We observe that the simulation results of the proposed algorithm follow the measured data closely in most cases. The proposed algorithm’s average error is 3.1161 dB, while the average prediction error of the traditional method is 6.1810 dB. Since this path is on the sea surface or land, the horizontal inhomogeneity of the duct layers on the Amsterdam–Burum path is obvious. Most of the predicted values of the traditional method are unstable since it neglects the range-dependant atmospheric conditions in near-shore areas.

Figure 10b shows the relationship between the prediction performance in Figure 10a and the standard deviation of the duct heights. As one can see, the standard deviation of the duct height on the Amsterdam–Burum path is smaller than that on the Goes–Burum path. When the standard deviation is more than 15 m, the sliced parabolic equation algorithm can achieve better performance.

## 5. Simulation Analysis

In this section, we further investigate the influence of different duct horizontal characteristics on the received signal strength.

### 5.1. Range-Dependent Evaporation Duct

In this subsection, the simulation results in horizontally inhomogeneous evaporation ducts are analyzed. The duct conditions are shown in Table 4. The propagation path is 210 km and the frequency is 3.5 GHz. The transmitter height is 15 m. The modified refractivity profiles can be calculated by the bulk method after obtaining the temperature, humidity, wind speed, and pressure. Under five different conditions, the evaporation duct heights (EDH) are 25 m, 32 m, 37 m, 19 m, and 16 m, respectively.

Assume that the 210 km path is divided into three slices. Case A is the horizontally homogeneous evaporation duct. The EDHs in the three slices are 25 m, 25 m, and 25 m. Case B and case C are simulated in horizontally inhomogeneous evaporation ducts. In case B, the EDH increases on the propagation path. The EDHs of the three slices are 25 m, 32 m, and 37 m. In case C, the EDH decreases on the propagation path. The EDHs of the three slices are 25 m, 19 m, and 16 m.

**Figure 11:**
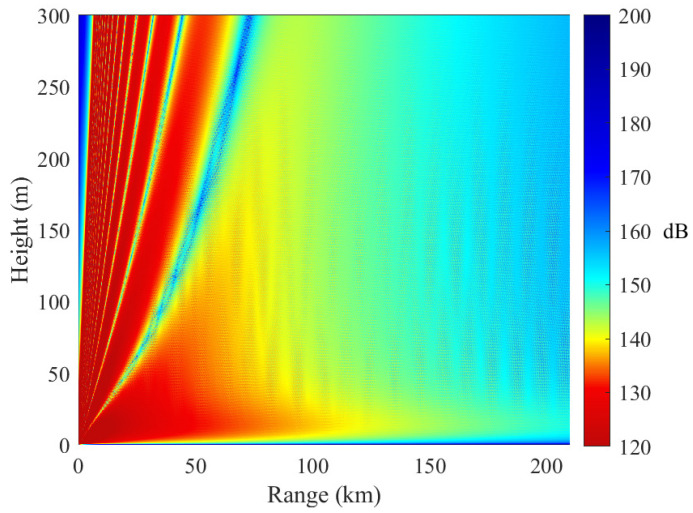
Path loss in a homogeneous evaporation duct. In case A, the EDHs of the three slices are 25 m, 25 m, and 25 m, respectively.

Figure 11 shows the electromagnetic wave propagation in a homogeneous evaporation duct. It is shown that the evaporation duct behaves similarly to a waveguide and can lead to decreased path loss. For example, the path loss is approximately 140 dB at 50 km when the receiving antenna height is 3 m. The path loss at 210 km is approximately 160 dB, which is only 20 dB higher than that at 50 km. If the receiving antenna is located outside the evaporation duct, then the path loss is approximately 20 dB higher than that inside the evaporation duct.

**Figure 12:**
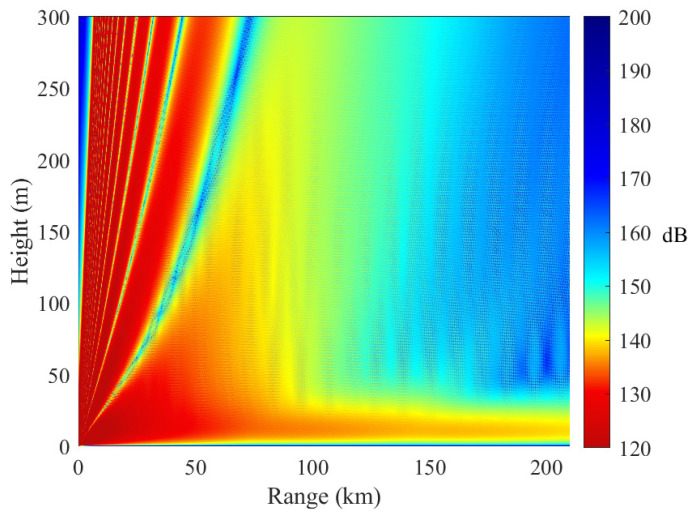
Path loss in horizontally inhomogeneous evaporation ducts. In case B, the EDHs of the three slices are 25 m, 32 m, and 37 m, respectively.

**Figure 13:**
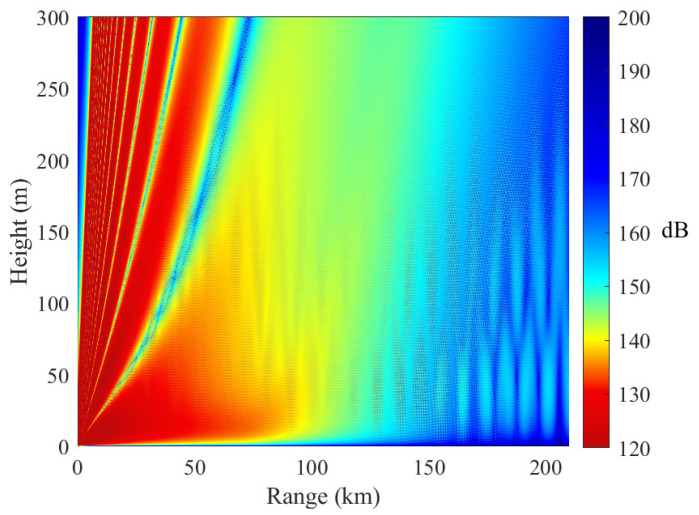
Path loss in horizontally inhomogeneous evaporation ducts. In case C, the EDHs of the three slices are 25 m, 19 m, and 16 m, respectively.

Figure 12 and Figure 13 show the electromagnetic wave propagation in inhomogeneous evaporation ducts. The propagation path is 210 km. The slice range is 70 km. The electromagnetic wave can be trapped in the two cases. However, the duct effects are quite different. The reasons are as follows.

In Figure 12, the EDH increases along the propagation path ([25 m, 32 m, 37 m]) and the trapping feature becomes stronger. The electromagnetic wave that departs from the evaporation duct returns to the duct due to the refractivity. From the figure, we observe that the path loss fades with respect to the vertical height. In other words, the antenna heights and evaporation duct heights are key parameters when evaporation ducts are used for long-distance maritime communications.

In Figure 13, the EDH decreases along the propagation path and the trapping feature becomes weaker. Therefore, some electromagnetic waves cannot be trapped and leave the duct layer. In the range of 140 km and 210 km, the duct effect is not obvious. The path loss is 177.80 dB at 210 km when the receiving antenna height is 3 m.

**Figure 14:**
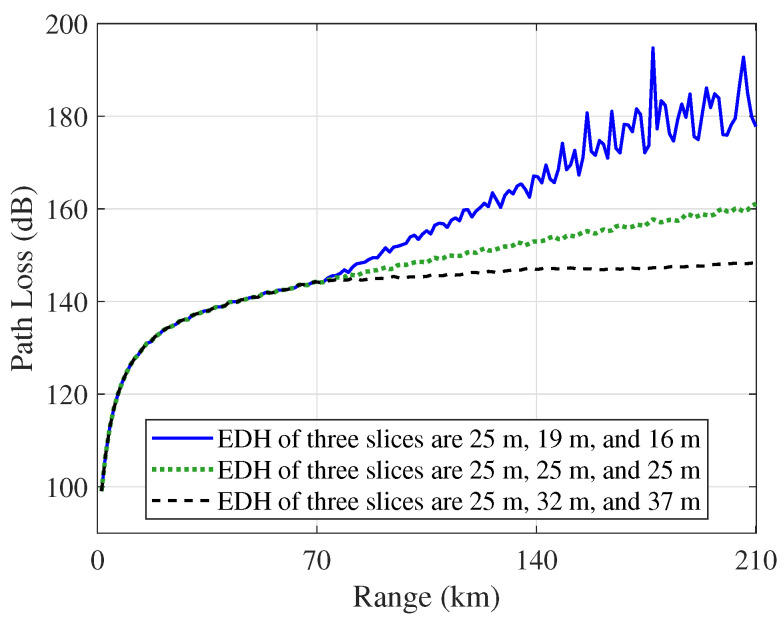
Path loss comparison in horizontally homogeneous and inhomogeneous evaporation ducts, when the height of the receiver is 3 m.

Figure 14 shows a comparison of the path loss in horizontally homogeneous and inhomogeneous evaporation ducts when the receiver height is 3 m. The numerical results indicate that the horizontal inhomogeneity of the evaporation duct exerts a larger influence on electromagnetic wave propagation. With increasing distance, the path loss difference gradually increases. There is a difference of approximately 30 dB in path loss between the two inhomogeneous cases. The simulation results show that the duct horizontal characteristics have a great impact on the received signal strength.

The above simulation results show that the duct effect of propagation from the low EDH to the high EDH is better than that under the opposite conditions. The radio propagation in a low evaporation duct height environment has a more obvious horizontally inhomogeneous influence.

### 5.2. Range-Dependent Elevated Duct

In this subsection, we evaluate the performance of the proposed algorithm in horizontally inhomogeneous elevated ducts. The simulation parameters are shown in Table 5. The propagation path is 210 km and the frequency is 3.5 GHz. Note that the transmitter height is 25 m. Assume that the path is divided into three slices. According to the structure of elevated ducts in Section 2, we set the base heights and top heights of elevated ducts, which are listed in Table 5. Without loss of generality, we assume that $\frac{dM}{dz}=-43$ M-unit/km.

**Figure 15:**
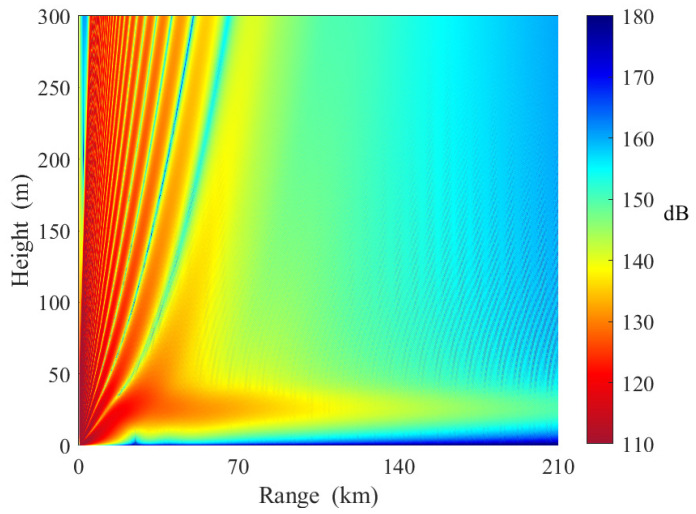
Path loss in horizontally homogeneous elevated ducts (Case D in Table 5).

Figure 15 shows the electromagnetic wave propagation in horizontally homogeneous elevated ducts. The base height and top height of the three slices are [20 m, 55 m], [20 m, 55 m], and [20 m, 55 m], respectively. From Figure 15, we can see the duct effects at heights between 20 and 55 m. When the receiving antenna height is less than 10 m, the path loss increases rapidly with distance. The reasons are as follows. Unlike evaporative ducts, there are standard refraction conditions between the elevated duct layer and the ground. Some electromagnetic waves cannot satisfy the angle of incidence conditions and leave the elevated duct.

**Figure 16:**
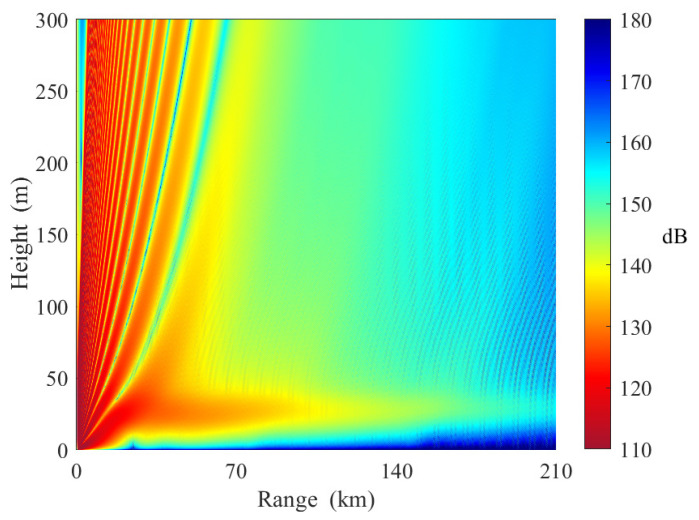
Path loss in horizontally inhomogeneous elevated ducts (Case E in Table 5).

In Figure 16, the strength of the elevated duct decreases along the path. The base height and top height of the three slices are [20 m, 55 m], [23 m, 55 m], and [27 m, 55 m], respectively. It can be observed that the signal can be trapped in elevated ducts in the first and second slices. When the receiving antenna height is 9 m, the path loss is approximately 156 dB at 140 km. However, the path loss increases rapidly over the third distance. The path loss is approximately 170 dB at 210 km. This is because the base height of the elevated duct in the third distance is higher than the transmitting antenna height.

**Figure 17:**
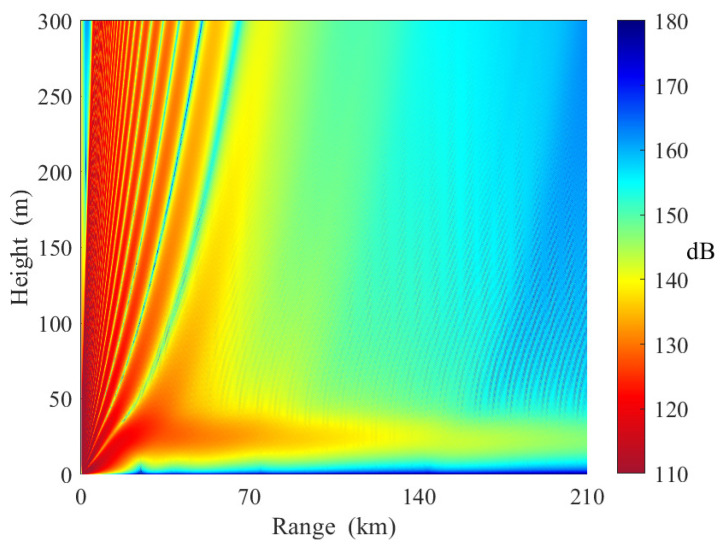
Path loss in horizontally inhomogeneous elevated ducts (Case F in Table 5).

Figure 17 shows the path loss distribution in horizontally inhomogeneous elevated ducts, where the conditions of elevated ducts improve along the path. The base height and top height of the three slices are [20 m, 55 m], [17 m, 55 m], and [13 m, 55 m], respectively. We can see that the electromagnetic wave can be trapped in elevated ducts along the entire path. Compared with Figure 15 and Figure 16, the duct effect is much stronger in the range of 140–210 km. When the receiving antenna height is 9 m, the path loss at 160 km is approximately 151 dB.

**Figure 18:**
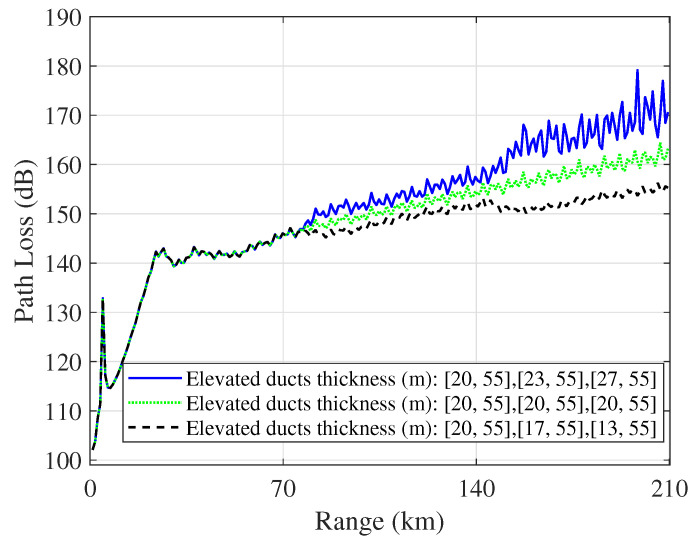
Path loss comparison in horizontally homogeneous or inhomogeneous elevated ducts when the height of the receiver is 9 m.

Figure 18 shows a comparison of the path loss in horizontally homogeneous and inhomogeneous elevated ducts when the receiver height is 9 m. With increasing distance, the path loss difference gradually increases. There is a difference of approximately 15 dB in path loss between the two inhomogeneous cases. Moreover, it is noteworthy that the fluctuation in path loss is obvious in all cases. Compared with evaporation ducts, the number of reflection paths increases due to the signals propagated inside the elevated duct and in the region between the duct and the sea surface.

In order to apply the algorithm better, we list the advantages and disadvantages of the proposed algorithm in Table 6. It is generic with the help of meteorological measurements. It can predict the propagation loss under range-dependent refractivity conditions. Future work can focus on sea surface roughness analysis under atmospheric duct conditions. It will be an important direction to exploit atmospheric ducts to improve the cell coverage in maritime scenarios, such as BS–ship and UAV–ship communications.

## 6. Conclusions

In this paper, we focused on the effect of horizontally inhomogeneous ducts on maritime communications from both theoretical and measurement perspectives. Through a thorough investigation and analysis of radio propagation in atmospheric ducts, we proposed a sliced parabolic equation algorithm to support the actual duct environment. Then, we derived the corresponding numerical solution and the approximate error expression. The proposed algorithm was verified by the 3.5 GHz long-distance radio measurement data. Results demonstrated that the proposed algorithm had better performance than the traditional parabolic equation method. We also provided the simulation results under duct conditions with different horizontal characteristics.

## Figures and Tables

**Table 1:** Details of the transmitter and receiver.

Location	Antenna Height	Frequency	Distance
Burum	6 m	-	-
Amsterdam	107 m	3.449001 GHz	138 km
Goes	75 m	3.449005 GHz	253 km

**Table 2:** The occurrence time of significant signal enhancements caused by atmospheric ducts.

Goes–Burum Path	Amsterdam–Burum Path
11 December 2013	11 December 2013
19 July 2014	30 April 2014
21 April 2015	7 June 2014
2 August 2015	1 August 2014
19 July 2016	10 August 2015
27 August 2016	7 September 2016

**Table 3:** Simulation parameters for the Goes–Burum link and Amsterdam–Burum link.

Parameters	Values
Frequency	3.45 GHz
Antenna height	75 m (Goes) and 107 m (Amsterdam)
Polarization	vertical
3 dB Beamwidth	8°
Elevation angle	0°
Max range	253 km
Max height	400 m
Range step size	80 m
Height step	1 m

**Table 4:** Simulation conditions for evaporation ducts.

Simulation Parameters	
Frequency	3.5 GHz
Antenna height	15 m
Polarization	Horizontal
3 dB Beamwidth	5°
Elevation angle	0°
Max range	0–210 km
Max height	0–300 m
**Meteorological Information**	
Wind speed	8 m/s
Height of wind speed	10 m
Temperature of air (Ta)	21, 21.5, 22, 20.5, 20 °C
Height of temperature	2 m
Relative humidity	67.8%
Height of relative humidity	2 m
Sea level pressure	1013.25 hPa
Sea surface temperature	20 °C
**Corresponding evaporation duct height**	
Evaporation duct height	25, 32, 37, 19, 16 m

**Table 5:** Simulation conditions for elevated ducts.

Simulation Parameters	
Frequency	3.5 GHz
Antenna height	25 m
Polarization	Horizontal
3 dB Beamwidth	5°
Elevation angle	0°
Max range	0–210 km
Max height	0–300 m
**Elevated duct heights of three slices** **[Base height (m), Top height (m)]**	
Case D	[20, 55], [20, 55], [20, 55]
Case E	[20, 55], [23, 55], [27, 55]
Case F	[20, 55], [17, 55], [13, 55]

**Table 6:** Advantages and disadvantages of the proposed algorithm.

Advantages	It supports range-dependent evaporation ducts and elevated ducts.
	It supports standard meteorological reanalysis data.
	It can support boundary condition setting.
	It can support calm sea surface conditions.
	Detailed feasibility analysis.
	Long-distance measurement verification at 3.5 GHz.
Disadvantages	It does not support detailed sea surface roughness analysis.
	It does not support millimeter-wave band.
	It does not support complex inland terrain.

## Data Availability

The meteorological data used in this study are available through the ERA5 hourly data on single levels website. The path loss data that support the findings of this study are available from the corresponding author upon reasonable request.
